# Tem­per­ature dependence in Bragg edge neutron transmission measurements

**DOI:** 10.1107/S1600576722006549

**Published:** 2022-07-30

**Authors:** Ala’a M. Al-Falahat, Nikolay Kardjilov, Robin Woracek, Mirko Boin, Henning Markötter, Luise Theil Kuhn, Malgorzata Makowska, Markus Strobl, Beate Pfretzschner, John Banhart, Ingo Manke

**Affiliations:** a Helmholtz-Zentrum Berlin für Materialien und Energie, Hahn-Meitner-Platz 1, Berlin 14109, Germany; b Mechanical Engineering Department, Faculty of Engineering, Mutah University, PO Box 7, Al-Karak 61710, Jordan; c Technische Universität Berlin, Strasse des 17 Juni 135, Berlin 10623, Germany; d European Spallation Source ERIC, PO Box 176, Lund 22100, Sweden; e Nuclear Physics Institute of the CAZ, Czech Republic; f Bundesanstalt für Materialforschung und -prüfung (BAM), Unter den Eichen 87, Berlin 12205, Germany; g Department of Energy Conversion and Storage, Technical University of Denmark, Fysikvej B310, Kongens Lyngby, 2000, Denmark; h Paul Scherrer Institut, WBBA/119, Villigen PSI, 5232, Switzerland; i Niels Bohr Institute, University of Copenhagen, Copenhagen, DK-2100, Denmark; Australian Nuclear Science and Technology Organisation, Lucas Heights, Australia

**Keywords:** neutron Bragg edge imaging, Debye–Waller factor, tem­per­ature-dependent neutron transmission, super martensitic stainless steel

## Abstract

A systematic study has been carried out to investigate the neutron transmission signal as a function of sample tem­per­ature. In particular, the experimentally de­ter­mined wavelength-dependent neutron attenuation spectra for a martensitic steel at tem­per­atures ranging from 21 to 700°C are com­pared with simulated data. A theoretical description that includes the Debye–Waller factor in order to describe the tem­per­ature influence on the neutron cross sections was im­plemented in the *nxsPlotter* software and used for the simulations. The analysis of the attenuation coefficients at varying tem­per­atures shows that the missing contributions due to elastic and inelastic scattering can be clearly distinguished: while the elastically scattered intensities decrease with higher tem­per­atures, the inelastically scattered intensities increase, and the two can be separated from each other by analysing unique sharp features in the form of Bragg edges. This study presents the first systematic approach to qu­antify this effect and can serve as a basis , for example, to correct measurements taken during *in situ* heat treatments, in many cases being a prerequisite for obtaining qu­anti­fiable results.

## Abstract 2

A systematic study was carried out to investigate the neutron transmission signal as a function of sample tem­per­ature. In particular, the experimentally determined wavelength-dependent neutron attenuation spectra for a martensitic steel at tem­per­atures ranging from 21 to 700°C are com­pared with simulated data.

## 1. Introduction

Neutron imaging has a broad field of applications ranging from materials and energy research to geology and plant science (Kardjilov *et al.*, 2018 ▸; Banhart, 2008 ▸). In recent years, many new neutron imaging techniques and modes have been introduced, and particularly the analysis of Bragg edges in wavelength-selective imaging has become of broad inter­est because it provides information about crystalline structures (*e.g.* Meggers *et al.*, 1994 ▸; Steuwer *et al.*, 2001 ▸, 2005 ▸; Vogel, 2000 ▸; Santi­steban *et al.*, 2002*a* ▸; Woracek *et al.*, 2018 ▸; Sato, 2018 ▸). The technique exploits variations in the transmitted neutron beam caused by scattered neutrons due to diffraction from crystal lattice sites. It can hence be very useful for investigating tem­per­ature-driven effects such as phase transformations in metallic/crystalline materials or reduction and oxidation processes. However, for qu­anti­tative data analysis it is necessary to take into consideration that the diffracted intensity depends on the tem­per­ature of the investigated material. The Debye–Waller factor describes the decrease of the elastically diffracted intensity caused by thermal vibrations of atoms at finite tem­per­atures (James, 1954 ▸; Warren, 1990 ▸; Pecharsky & Zavalij, 2009 ▸).

The effect of thermal vibrations of atoms is readily observed in the transmission spectrum of cold and thermal neutrons and has been shown several times (*e.g.* Priesmeyer *et al.*, 1999 ▸; Sato, 2018 ▸; Bourke *et al.*, 1996 ▸). A recent study demonstrated, for example, how one can effectively distinguish between ice, super-cooled water and water, where a higher mobility of protons causes an increase of inelastic scattering com­ponents (Siegwart *et al.*, 2019 ▸). However, up to now, no qu­anti­tative experimental strategies have been presented that exploit tem­per­ature-driven spectral variations of the measured attenuation coefficients in crystalline materials. Changes of Bragg edge heights were qualitatively shown and procedures suggested to use ‘the value of the total cross section for wavelengths beyond the first Bragg edge to define the tem­per­ature of the sample’ (Santi­steban *et al.*, 2002*b* ▸), while shifts of the Bragg edge position due to thermal expansion were examined, for example, by Vogel (2000 ▸) and Song *et al.* (2017 ▸). Significant attention has been given to exploiting the Doppler broadening in neutron resonance absorption imaging (Priesmeyer *et al.*, 1999 ▸; Tremsin *et al.*, 2016 ▸; Sato, 2018 ▸), which can be used for remote tem­per­ature measurements (using epithermal and inter­mediate neutrons in the energy range between ∼1 and ∼1000 eV). Knowledge of the neutron total cross sections of materials is obviously of inter­est beyond imaging, and extensive experimental data tables have been com­piled (*e.g.* Sears, 1992 ▸; Robledo *et al.*, 2020 ▸; Dawidowski *et al.*, 2013 ▸).

Wavelength-selective neutron imaging in the thermal and cold range is routinely applied to polycrystalline materials, where diffraction contrast (Woracek *et al.*, 2018 ▸) leads to Bragg edges in the transmission spectra that are the footprint of the crystalline structure (*e.g.* Xie *et al.*, 2018 ▸; Song *et al.*, 2017 ▸) and can be used to study applied and residual strain and stress (*e.g.* Sun *et al.*, 2018 ▸; Gregg *et al.*, 2017 ▸; Hendriks *et al.*, 2017 ▸; Woracek *et al.*, 2011 ▸), textures (*e.g.* Oikawa *et al.*, 2018 ▸; Santi­steban *et al.*, 2006 ▸), effects of grain sizes (*e.g.* Oikawa *et al.*, 2017 ▸; Sato, 2018 ▸), and crystalline phase identification and qu­anti­fication of *in situ* phase transition studies (*e.g.* Makowska *et al.*, 2015*a* ▸, 2017 ▸, 2018 ▸; Steuwer *et al.*, 2004 ▸; Bourke *et al.*, 1996 ▸; Huang *et al.*, 2007 ▸; Woracek *et al.*, 2014 ▸; Tran *et al.*, 2021 ▸).

In order to qu­anti­tatively describe the wavelength-dependent inter­action between neutrons and matter, neutron cross sections (conveniently expressed in terms of barns (1 barn = 10^−28^ m^2^) are utilized, which represent the likelihood of inter­action between an incident neutron and a target nucleus.

In the presented study, a series of wavelength-selective *in situ* neutron imaging experiments were performed, where a sample of super martensitic stainless steel (SMSS) was sequentially heated inside a furnace from 21°C up to 700°C. The changes of the transmitted intensity were analysed in order to investigate the effect of thermal expansion and thermal vibration on the neutron cross sections, and consequently on the attenuation coefficient of the selected steel. The software program *nxsPlotter* (Boin, 2012 ▸) was used to calculate the total cross sections of crystalline materials as a function of the neutron wavelength, and the output is herein com­pared with the experimental results. The contributions of the scattering mechanisms were evaluated and enable us to separate elastic from inelastic scattering in the transmission spectrum. SMSS was chosen with the objective of an overarching project examining the phase transformation kinetics of samples with and without the presence of hydrogen. The herein presented work forms the prerequisite to perform such a qu­anti­tative examination, which will be disseminated in a separate publication.

## 2. Theory

### 2.1. Debye–Waller factor

The Debye tem­per­ature θ_D_ of a bulk solid-state crystalline material is a measure of the rigidity of the bonds inside the crystal where the movement of one atom about its site makes the neighbouring atoms react to this motion. This results in the vibration of many atoms, which collectively spreads throughout the crystal (Yates, 2015 ▸). Each type of crystal lattice has its own mode of oscillation called regular mode, and therefore the overall collective oscillation movement of the lattice is a combination of many regular modes (Owens & Poole, 2008 ▸). The Debye–Waller factor (here applied by an isotropic displacement factor *B*_iso_) is connected to the Debye tem­per­ature as shown in the supporting information.

**Figure 1:**
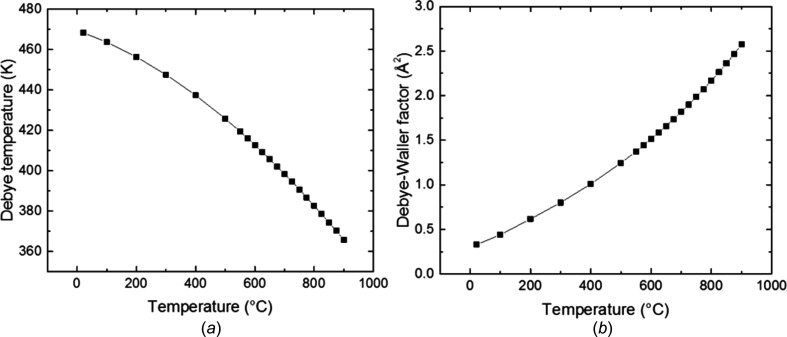
(*a*) The calculated Debye tem­per­ature θ_D_ as a function of sample tem­per­ature and (*b*) the calculated Debye–Waller factor *B*_iso_ for the same sample (super martensitic stainless steel).

Calculated values of the Debye–Waller factor *B*_iso_ and the Debye tem­per­ature θ_D_ at different tem­per­atures for the body-centred cubic (b.c.c.) steel are shown in Fig. 1 ▸. From these calculations, a Debye tem­per­ature of 468.3 K is found at 294 K (21°C) sample tem­per­ature and of about 412.6 K when raising the tem­per­ature *T* to 873 K (600°C). In contrast, the inverse trend is seen for the Debye–Waller factor, which starts at 0.33 Å^2^ at 294 K and increases to 1.5 Å^2^ at 873 K.

### 2.2. Scattering cross sections

To understand the tem­per­ature dependence of the Debye–Waller factor in polycrystalline materials, the total microscopic neutron cross section of an isotope is calculated, which is the cross section σ_tot_ for an incoming neutron to inter­act with the material. It is given by its incoherent (σ_incoh_) and coherent (σ_coh_) scattering, as well as its absorption (σ_abs_) contributions (Vogel, 2000 ▸; Granada, 1984 ▸):

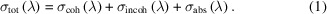




**Figure 2:**
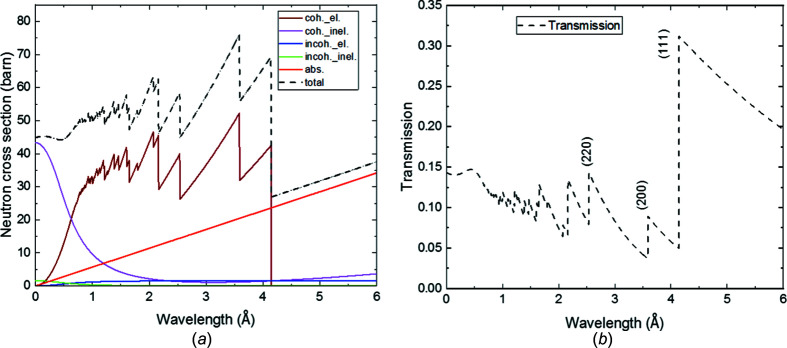
Theoretical neutron cross sections (*a*) for an f.c.c. iron unit cell and (*b*) for an ideal transmission spectrum through 2 cm thick f.c.c. iron calculated by the *nxsPlotter* software (Boin, 2012 ▸).

The overall formulation of the total cross section is given by Granada (1984 ▸) and Vogel (2000 ▸), and is presented in detail in the supporting information. This formulation was later applied by Boin (2012 ▸) in the *nxsPlotter* software for cross section calculations, which we use in this work. In this model, all types of neutron scattering of the material at different Debye–Waller tem­per­atures as well as varying sample tem­per­atures are included. This is given by its incoherent and coherent (elastic and inelastic) scattering, as well as its absorption contribution. An example for face-centred cubic (f.c.c.) iron is presented in Fig. 2 ▸ (note that the f.c.c. structure corresponds to the austenitic phase in the SMSS investigated herein, whereas the martensitic phase exhibits a b.c.c. structure). This calculation allows us to study the effect of the Debye–Waller factor on the total neutron cross section. Note that the formulation assumes the crystal to be a powder-like assembly of small crystal grains of random orientation.

The sum of the contributions from absorption and scattering is used to com­pute the transmission shown in Fig. 2 ▸(*b*) via the attenuation coefficient μ as described by Beer–Lambert’s law:

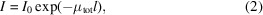

where *I*_0_ is the intensity of the incident beam and *I* is the intensity that is detected, while *l* is the sample thickness. The linear attenuation coefficient μ_tot_ is defined by the particle density *N* and the total microscopic cross section σ_tot_ as (Binder, 1970 ▸; Steuwer *et al.*, 2005 ▸)

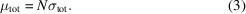




## 3. Experimental procedure

**Figure 3:**
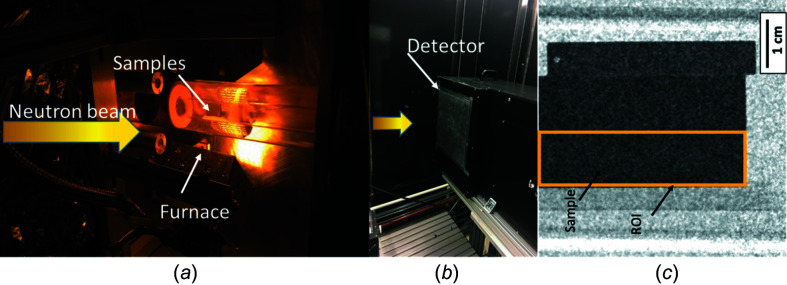
The experimental setup with (*a*) a furnace (placed in front of the detector, which cannot be seen here), with three samples stacked on top one another, and (*b*) a detector (shown without the furnace for better visibility). (*c*) The radiography image of the sample inside the furnace taken at 4.02 Å neutron wavelength [note that there are three samples, but this study focuses only on the sample that is highlighted by an orange rectangle (ROI)].

The neutron imaging beamline CONRAD-2 (Kardjilov *et al.*, 2016 ▸) was used to conduct a series of neutron wavelength scans while the sample was heated in a furnace to different tem­per­atures, namely, 21, 200, 400, 500, 600 and 700°C. The sample under investigation, a super martensitic stainless steel (see Table 1 ▸), had physical dimensions of 33.1 × 9.3 × 5.6 mm (length × height × thickness). It was heated by two IR heaters, each of which was equipped with six halogen quartz lamps and water-cooled polished aluminium reflectors which illuminate an area of 100 × 75 mm (*i.e.* much larger than the sample). More technical information about the setup can be found elsewhere (Makowska *et al.*, 2015*b* ▸). The sample was kept inside the innermost of two concentrically arranged quartz tubes, as shown in Fig. 3 ▸. The tube was sealed at the ends and the sample tem­per­ature was measured with a thermocouple attached to it and controlled remotely. The sample was heated to the target tem­per­atures at a rate of 50°C min^−1^.

A scintillator-camera-based detector system was employed for this experiment (scintillator: 200 µm ^6^LiZnS:Ag; camera: CCD Andor DW436 2048 × 2048 pixel, pixel size: 48 µm), as described by Kardjilov *et al.* (2016 ▸). With an exposure time for each image of 60 s, a wavelength scan from 3.5 to 4.2 Å, and in some tem­per­ature cases up to 4.4 Å, with steps of 0.02 Å, was performed, where for each step the transmission through the sample was measured. The monochromatic beam was achieved by a tunable double-crystal monochromator with a resolution (Δλ/λ) of ∼1.36% for the used crystal mosaicity of 0.8° (Al-Falahat *et al.*, 2019 ▸). The transmission was obtained by normalizing the images of the sample by open beam images (*i.e.* no sample in the beam), as well as dark field images (*i.e.* no neutron beam). Thus, any beam or detector inhomogeneity was corrected. The analysis of the images was accom­plished using the software *ImageJ* (Abràmoff *et al.*, 2004 ▸). The wavelength-dependent neutron transmission through the sample can be plotted for each pixel of the detector. However, for the presented results, the overall intensity for a region slightly smaller than the sample itself [region of inter­est, ROI; com­pare Fig. 3 ▸(*b*)] was selected in order to ensure that any possible surface effects are excluded while maximizing the signal-to-noise ratio.

## 4. Results and discussion

**Figure 4:**
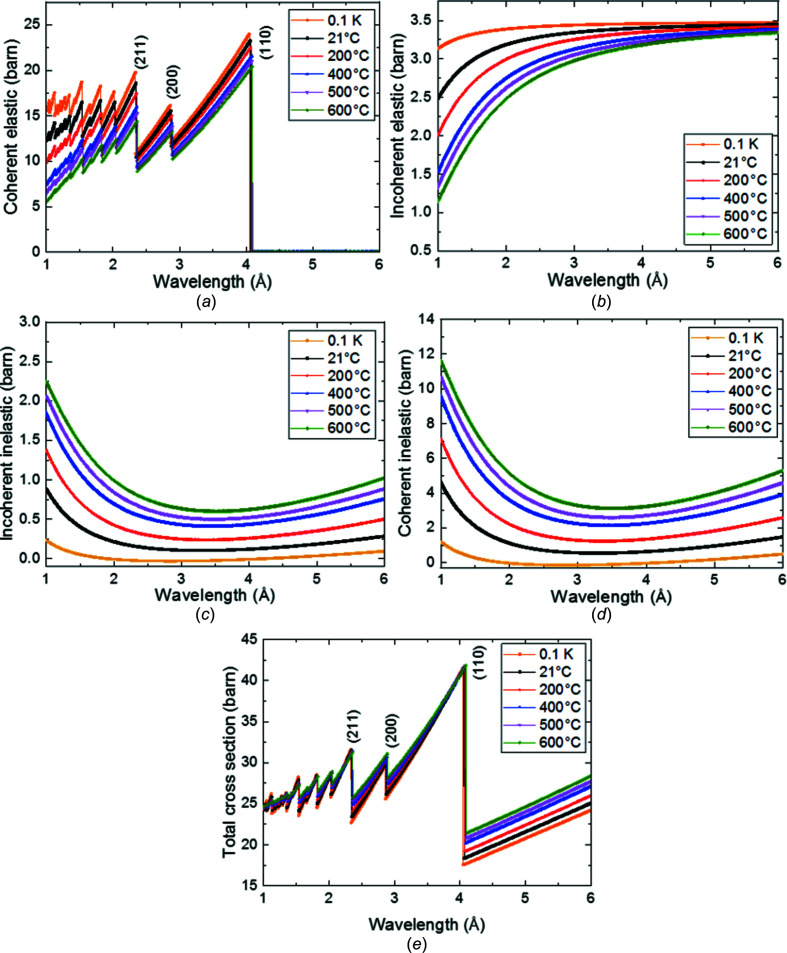
Calculated scattering contributions for the investigated super martensitic stainless steel (b.c.c.) at different tem­per­atures: (*a*) coherent elastic, (*b*) incoherent elastic, (*c*) incoherent inelastic, (*d*) coherent inelastic and (*e*) total neutron scattering cross section being the experimentally observed property.

Fig. 4 ▸ shows the effect of the sample tem­per­ature for a range between 21 and 600°C on the neutron cross section as cal­cul­ated using the program *nxsPlotter*, on the basis of input parameters such as sample tem­per­ature, Debye tem­per­atures and crystal lattice constants. The coherent elastic scattering cross section [Fig. 4 ▸(*a*)] and the incoherent elastic scattering cross section [Fig. 4 ▸(*b*)] both decrease with higher tem­per­atures, where the decrease is more pronounced for lower wavelengths.

According to Bragg’s law, coherent elastic scattering from a particular lattice plane family *hkl* cannot occur for neutrons with wavelengths longer than λ_max_, which corresponds to twice the *d* spacing of the specific lattice plane family. Hence the sudden increase of transmission causes the characteristic Bragg edge(s). Because an atom in a crystal is never at rest but oscillates around its average position, and by using the Debye model for crystal vibrations, one can show that the coherent elastic scattering amplitude is reduced with higher tem­per­atures, as depicted in Fig. 4 ▸(*a*).

**Figure 5:**
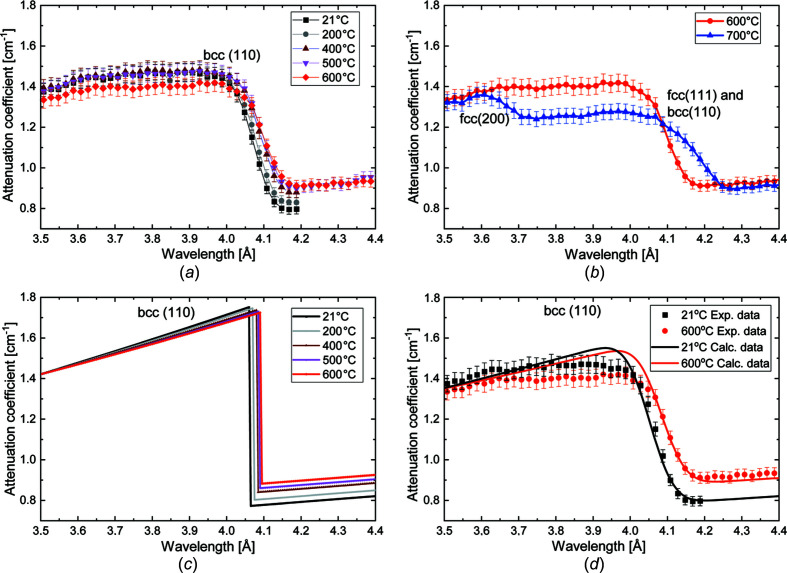
Calculated and measured wavelength-dependent attenuation coefficients. (*a*) Measurements at 21, 200, 400, 500 and 600°C. (*b*) Measurements at 600 and 700°C revealing the progression of the phase transformation from b.c.c. to f.c.c. at 700°C. (*c*) Values calculated using the software *nxsPlotter* for the single b.c.c. phase. (*d*) Comparison between experimental data measured at 21 and 600°C, taken from (*a*), and the corresponding calculated values given in (*c*). The calculated data were smeared by convolution with the wavelength resolution function of the double-crystal monochromator.

Moreover, at elevated tem­per­atures, the *d* spacings increase due to thermal expansion and hence the position of the Bragg edge(s) ‘shift’ according to that expansion. For the Bragg edge corresponding to b.c.c. (110), this shift can be calculated as 0.013, 0.027 and 0.042 Å at 200, 400 and 600°C, respectively. These values are calculated according to the lattice parameter accounting for thermal expansion as reported by Christien *et al.* (2013 ▸) for an alloy com­position similar to that investigated herein. The shifts of the Bragg edge positions are seen best when the wavelength scale is magnified, such as in Fig. 5 ▸(*c*).

As shown in Fig. 4 ▸(*b*), the neutron scattering cross section of the disordered (incoherent) part of the elastic scattering decreases with tem­per­ature in a similar way to the ordered (coherent) com­ponent. Moreover, the decrease is much more substantial for lower wavelengths and the difference is minimal for longer wavelengths. In our case, the incoherent part of the cross section actually provides a nearly constant contribution of 3.3 barns for 21°C at all wavelengths (between 1 and 6 Å) and is approximately the same value for all tem­per­atures at 6 Å.

The inelastic scattering contributions are shown in Figs. 4 ▸(*c*) and 4(*d*). With increasing thermal motion, inelastic scattering becomes more and more prominent. The cross section values of the coherent inelastic scattering part increase most [Fig. 4 ▸(*d*)], for example, from about 1 to 4 barns when going from 21 to 600°C, but the incoherent inelastic part [Fig. 4 ▸(*c*)] also increases from about 0.012 to 0.588 barns for the same tem­per­ature increase (in both cases for a wavelength of 4.2 Å). For this wavelength, the tem­per­ature effect in absolute numbers is largest for coherent inelastic scattering, featuring an increase of 3.4 barns of cross section com­pared with 0.58 barns for inelastic incoherent scattering. For other wavelengths in this regime, the effect is similar.

This reversed inelastic scattering intensity [Figs. 4 ▸(*c*) and 4(*d*)] com­pared with the elastic scattering intensity [Figs. 4 ▸(*a*) and 4(*b*)] manifests itself in such a way that there is almost no observable difference in the total cross section [Fig. 4 ▸(*e*)] right before every Bragg edge. However, right after every Bragg edge, the difference of total cross section is significant and dominated by the contributions due to inelastic scattering. In addition, the total neutron cross sections above the Bragg edge cut-off are proportional to the sample tem­per­atures, with total neutron cross sections of about 18.6, 19.3, 20.7, 21.3 and 21.7 barns at 4.2 Å at tem­per­atures of 21, 200, 400, 500 and 600°C, respectively, as seen in Fig. 4 ▸(*e*).

Measured transmission spectra for a wavelength range between 3.5 to 4.4 Å are shown in Fig. 5 ▸, depicting the wavelength-dependent attenuation coefficients around the Bragg edge corresponding to b.c.c. (110). The attenuation coefficients at a wavelength of 4.2 Å, as seen in Fig. 5 ▸(*a*), show a notable increase during heat treatment from 21 to 600°C from about 0.8 to 0.95 cm^−1^, respectively. The changes of the measured attenuation during heating before the Bragg cut-off are rather small (and range between 1.46 and 1.39 cm^−1^ at 3.9 Å). These observations agree well with the trend observed in the calculated results [Fig. 5 ▸(*c*)]. The wavelength-dependent attenuation coefficients measured at 600 and 700°C presented in Fig. 5 ▸(*b*) show that austenitization has clearly progressed at 700°C. This can be concluded from the Bragg edge appearing at ∼3.6 Å, corresponding to the f.c.c. (200) lattice plane family. Correspondingly, the b.c.c. (110) Bragg edge at ∼4.1 Å is fading, while f.c.c. (200) becomes more pronounced.

Fig. 5 ▸(*c*) shows calculated attenuation coefficients of various tem­per­atures. The difference just before the Bragg edge is rather small (with a decrease from 1.75 cm^−1^ at 21°C to 1.71 cm^−1^ at 600°C), whereas it is much more pronounced after the Bragg edge (with an increase from 0.78 cm^−1^ at 21°C to 0.88 cm^−1^ at 600°C), which has already been explained above by the differences of the inelastic scattering cross sections. The figure also shows the shift of the Bragg edge due to the thermal expansion, and the shift by approximately 0.040 Å when heating from 21 to 600°C agrees well with the experimentally observed data depicted in Fig. 5 ▸(*a*).

In Fig. 5 ▸(*d*), the measured and calculated attenuation coefficients are com­pared. The calculated curves were convoluted with the wavelength resolution function of the double-crystal monochromator [Gaussian, FWHM = 0.05 Å – defined for a wavelength resolution (Δλ/λ) of ∼1.36% at λ = 4 Å] (*e.g.* Boin, 2012 ▸; Al-Falahat *et al.*, 2019 ▸). The error bars correspond to the standard deviation of 3% determined by the integral intensity measurements in the ROI, as shown in Fig. 3 ▸(*c*). In addition, the influence of the tem­per­ature rises from 21 to 600°C in both the measured and calculated attenuation coefficients are clearly seen at 4.2 Å, showing an increase of the attenuation coefficient from about 0.80 to 0.91 cm^−1^.

**Figure 6:**
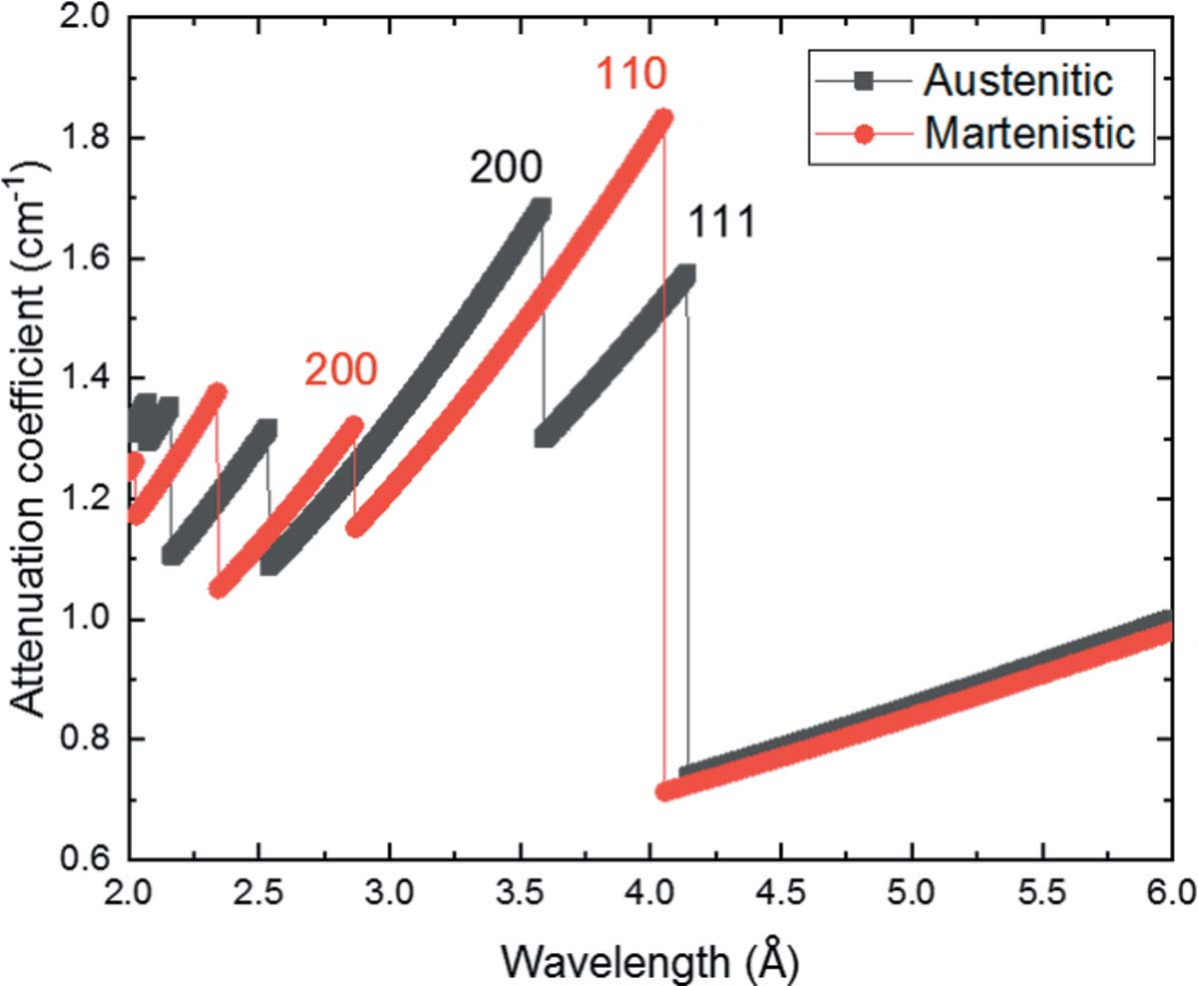
Comparisons between the attenuation coefficient of the martensitic structure (b.c.c.) and the austenitic structure (f.c.c.) at 21°C as calculated by the software *nxsPlotter*.

Another observation that can be made in Fig. 5 ▸(*d*) relates to the fact that the measured attenuation coefficient before the Bragg cut-off (between ∼3.7 and ∼4.0 Å) at 600°C decreases slightly more than what can be expected from the calculated values. While the experimental error margin could be one reason for this observation, another reason could be that the transformation from martensite to austenite is already starting at this tem­per­ature, since the neutron attenuation by martensite is larger than that by austenite within the wavelength range between 3.6 and 4.0 Å, as shown in Fig. 6 ▸. However, the f.c.c. (200) Bragg edge is not yet discernible and hence this explanation is still speculative.

**Figure 7:**
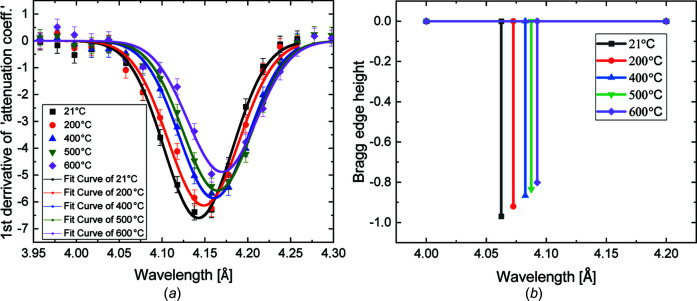
Bragg edge height of b.c.c. (110) for the five different tem­per­atures (21, 200, 400, 500 and 600°C) by (*a*) derivative and Gaussian fit of the measured Bragg edge, and (*b*) values of the theoretical Bragg edge heights based on Fig. 5 ▸(*c*). The distinct shift of the position of the Bragg edge is due to the thermal expansion.

In order to qu­anti­tatively describe the observed effects in the measurements and calculations, the Bragg edge position is extracted from the attenuation coefficient spectrum using nonlinear least-squares fitting. The derivative of the attenuation spectrum is com­puted and a Gaussian fit is applied [see Fig. 7 ▸(*a*)]. In order to minimize the influence of the subjective factor in the determination of the position of the Bragg edge (*e.g.* where the Bragg edge starts, where it ends, offset determination and so on), we decided to use a fitting procedure to derive this parameter. The Bragg edge height for the calculated Bragg edges is determined by subtracting the attenuation coefficient values before and after the Bragg edge. The corresponding values are shown in Fig. 7 ▸(*b*) and Table 2 ▸.

The error estimates determined from the least-squares fit are obtained for the five Bragg edge derivatives by

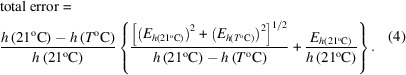

Here, *E_h_* is the error of the height at a selected tem­per­ature. In order to calculate the relative decrease of Bragg edge height, the measured heights must be normalized with respect either to the initial height or to the final height. The value at 21°C is taken as the initial height *h*(21°C) of the Bragg edge. The relative decrease of the Bragg edge height is calculated using equation (5), for both experiment and calculation, as shown in Table 2 ▸:

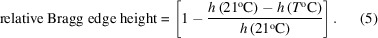




**Figure 8:**
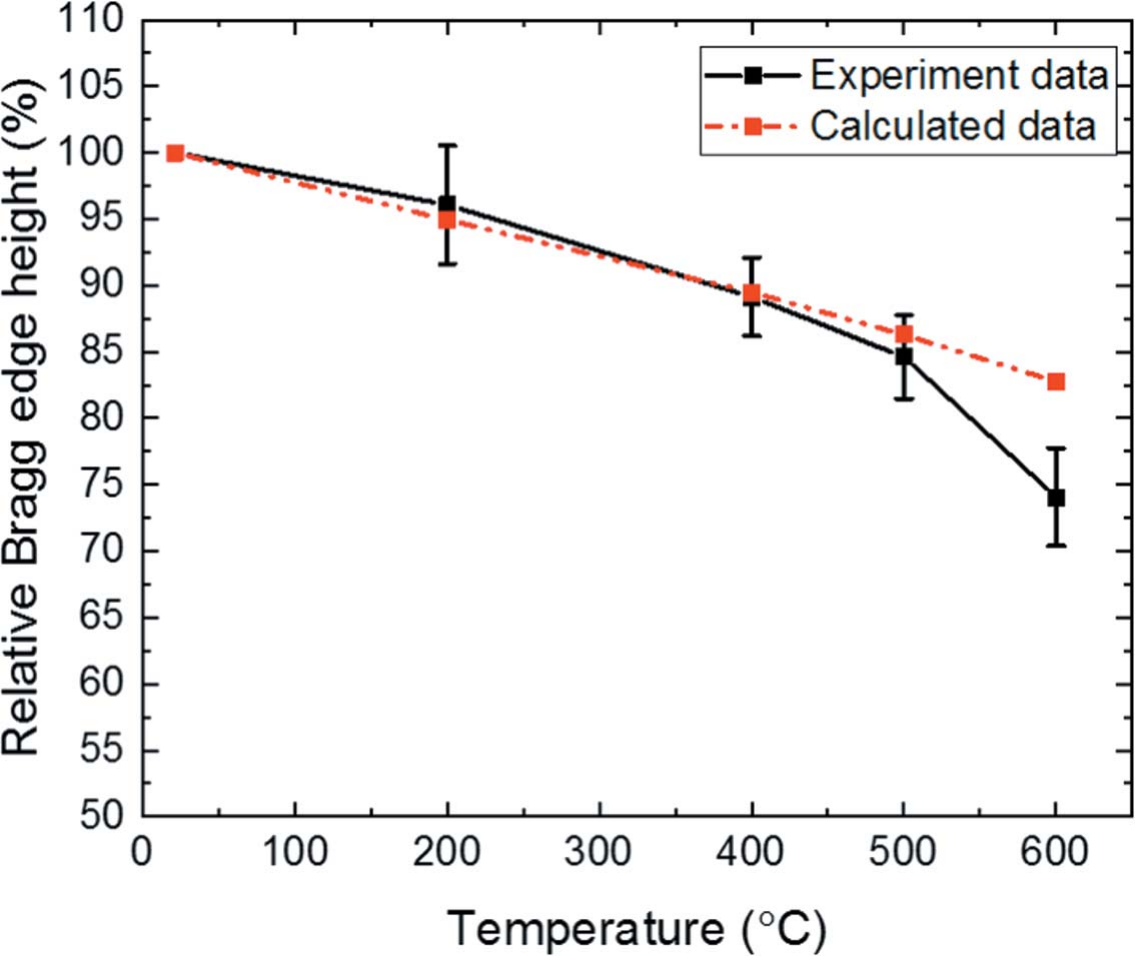
Relative decrease of the heights of the b.c.c. (110) Bragg edge as a function of tem­per­ature as calculated and measured. At 600°C the experimental value is ∼33% below the calculation; one possible explanation could be an onset of phase transformation to austenite. Error bars indicate the total standard deviation calculated from the relative Bragg edge height.

The influence of the thermal vibrations of atoms with increasing tem­per­ature can be observed in both the measured and the calculated data by a decrease of the Bragg edge height, as can be seen for the calculated and measured data in Fig. 8 ▸.

As a result, the decreasing height of the b.c.c. (110) Bragg edge com­pared with the data taken at 21°C can be determined from the experiment to be 4% at 200°C, 11% at 400°C and 15% at 500°C, as can be seen in Fig. 8 ▸ and Table 2 ▸, and can be fully attributed to the tem­per­ature dependence of the scattered intensity. This trend, caused by the tem­per­ature rise, is in good agreement with the calculated results and as herein described by the Debye–Waller factor.

Only for 600°C is some noticeable discrepancy observed. This difference may be attributed to the appearance of austenite, which starts to form during heating while the volume fraction of the martensitic phase is reduced (Christien *et al.*, 2013 ▸). In this case, there would be a superimposition of two effects that result in smaller Bragg edge heights: the scattering intensity described by the Debye–Waller factor and the smaller austenitic Bragg edge, f.c.c. (111), com­pared with the martensitic Bragg edge, b.c.c. (110).

## 5. Conclusions

The influence of thermal effects on the transmission spectra has been evaluated by carrying out neutron attenuation measurements of a martensitic steel sample at different tem­per­atures. Experimental data were com­pared with calculations based on the *nxsPlotter* library, where the Debye–Waller factor has been implemented to describe the observed effects of varying intensities. The calculated results show, as expected, that thermally induced vibrations affect all contributions to neutron scattering: with increasing sample tem­per­ature, the elastic neutron cross section decreases and the inelastic scattering cross section increases.

The Bragg edge height was analysed and, with the help of the calculated results obtained by *nxsPlotter*, we showed that it allows the scattering contributions due to coherent elastic scattering to be separated from the other scattering contributions. While the Bragg edge height itself is determined by the coherent elastic scattering contribution, both the coherent elastic scattering and incoherent elastic scattering cross sections are almost constant for wavelengths after the Bragg cut-off of the material, as is evident from the simulations. This means that experimentally determined differences after the Bragg cut-off can be attributed to inelastic scattering processes. A significant reduction in Bragg edge height was observed as a function of tem­per­ature. The analysis of the com­plete attenuation spectra showed that the Bragg edge varies in height for two reasons in our study: the scattered intensity decreases (*i.e.* smaller Bragg edges) with increasing tem­per­atures (as described by the Debye–Waller factor) and the onset of the phase transformation that reduces the volume fraction of the martensitic phase. In the investigated martensitic steel, the Bragg edge height reduction up to 500°C can be fully attributed to differences described by the Debye–Waller factor. The theoretical and experimental data are in very good agreement up to 500°C. At 700°C, the phase transformation from martensite to austenite is evident by the formation of the f.c.c. Bragg edges. The experimental data at 600°C deviate more strongly from the calculations, and one possible explanation is that the phase transformation has already started. If this is the case, then the herein utilized analysis would provide a high sensitivity for observing the onset of a phase transformation even before Bragg edges become visible.

The observed effect is notable with a 15% change of attenuation coefficient (Bragg edge height) at 500°C. If this effect is not taken into account properly, wrong inter­pretations of *in situ* heating/cooling experiments could be the consequence. Contrary to the conclusion drawn by Song *et al.* (2017 ▸), who stated that the Bragg edge ‘height reduction is believed to be caused by the grain growth’, we have demonstrated that the height reduction can be attributed to changes of the coherent elastic scattering contribution due to thermal motion and moreover that the differences after the Bragg cut-off can be attributed to the changing inelastic scattering contributions due to thermally induced lattice vibrations. If additional effects are to be investigated under *in situ* heating/cooling, such as grain growth (Song *et al.*, 2017 ▸), hydrogen effusion (Beyer *et al.*, 2011 ▸) or phase transformations (Dabah *et al.*, 2017 ▸), corresponding corrections for the tem­per­ature dependence are mandatory. This study can be used as a starting point for other researchers to introduce appropriate corrections in future experiments.

This study supports, as already suggested by Santi­steban *et al.* (2002*b* ▸), that the spectrum after the Bragg cut-off could be used to probe the tem­per­ature of the sample. Here it should be pointed out that future analysis can also be used, for example, to exploit qu­anti­fication of thermal diffuse scattering in transmission images or to utilize the region beyond the Bragg cut-off for restricted fitting in order to perform phase qu­anti­fication as it does not suffer from texture effects (Steuwer *et al.*, 2005 ▸). This work may even pave the way for wavelength-resolved neutron imaging to efficiently exploit effects related to inelastic scattering in general, as has only recently been started (Siegwart *et al.*, 2019 ▸).

The *nxsPlotter* software (Boin, 2012 ▸) has been updated as part of this work and now includes the possibility to simulate more com­plex alloying com­positions (as investigated in this work and typical for most metallic alloys) to obtain realistic attenuation coefficients. Ongoing work is concerned with imple­menting these routines into the neutron ray tracing software package *McStas* (Lefmann & Nielsen, 1999 ▸), which allows the prediction of the transmission spectra which will be obtained due to the instrument resolution function. This tool is openly available and supports the qu­anti­tative exploitation of wavelength-dependent transmission spectra, especially in view of the new imaging beamlines at the powerful spallation sources, *e.g.* IMAT@ISIS, ODIN@ESS, RADEN@J-Parc and VENUS@SNS (Kockelmann *et al.*, 2015 ▸; Shinohara & Kai, 2015 ▸; Bilheux *et al.*, 2015 ▸; Strobl, 2015 ▸).

## 6. Related literature

The following are cited in the supporting information: Alers (1965 ▸); Balaguru & Jeyaprakash (2015 ▸); Dinnebier & Billinge (2008 ▸); Ghosh & Olson (2002 ▸); Herbstein (1961 ▸); Miettinen & Louhenkilpi (1994 ▸); Schopper (2000 ▸); Sirdeshmukh *et al.* (2006 ▸); Terasaki *et al.* (2011 ▸); Willis & Carlile (2017 ▸); Windsor (1981 ▸); Yang *et al.* (2006 ▸).

## Supplementary Material

Supporting information. DOI: 10.1107/S1600576722006549/in5052sup1.pdf

## Figures and Tables

**Table 1:** Chemical com­position of the super martensitic stainless steel in wt%

C	Mn	Si	Co	Ni	Cr	Mo	Fe
0.006	1.87	0.294	0.475	6.498	11.65	2.33	76.88

**Table 2:** Height and relative height of the (110) Bragg edge as a function of tem­per­ature as measured and calculated The FWHM of the fitted data is 0.09 for all tem­per­atures.

Tem­per­ature (°C)	Bragg edge height experiment	Bragg edge height calculated	Relative Bragg edge height experiment (%)	Relative Bragg edge height calculated (%)
21	−0.663 ± 0.014	−0.969	100	100
200	−0.637 ± 0.026	−0.920	96 ± 4	95
400	−0.591 ± 0.014	−0.867	89 ± 3	89
500	−0.561 ± 0.016	−0.836	85 ± 3	86
600	−0.491 ± 0.020	−0.802	74 ± 4	83
