# Supplementary material for: Tem­per­ature dependence in Bragg edge neutron transmission measurements
Source: J Appl Crystallogr. 2022 Jul 30;55(Pt 4):919–28. doi: 10.1107/S1600576722006549 (PMC9348867; doi:10.1107/S1600576722006549)
Supplement: Supplementary file 1 [file j-55-00919-sup1.pdf]

JOURNAL OF APPLIED CRYSTALLOGRAPHY

Volume 55 (2022)

Supporting information for article:

Temperature dependence in Bragg edge neutron transmission measurements

Ala'a M. Al-Falahat, Nikolay Kardjilov, Robin Woracek, Mirko Boin, Henning Marktter, Luise Theil Kuhn, Malgorzata Makowska, Markus Strobl, Beate Pfretzschner, John Banhart and Ingo Manke

1. Theory

1.1. Debye-Waller factor

The Debye temperature relates to the binding force between atoms and can be utilized to signify the properties of many materials, which include thermal vibrations of atoms or phase transitions (Yang *et al.*, 2006). It reflects the crystal's highest vibration mode and is given by $\theta_D = h\nu_D/k_B$, where ν_D is the characteristic Debye frequency, k_B Boltzmann's and h Planck's constant, respectively (Balaguru & Jeyaprakash, 2015).

The Debye-Waller factor (here applied by an isotropic displacement factor B_{iso}) is connected to the Debye temperature through the following equation (Sirdeshmukh *et al.*, 2006):

$$B_{\mathrm{iso}} = \frac{3h^2\, \varphi_1(\mathrm{x})}{M\, k_B \theta_D}, \qquad (1)$$

where M is the mass of the scattering nucleus, $\varphi_1(\mathrm{x})$ is the Debye integral function with its argument $\mathrm{x} = T/\theta_D$ and T the sample temperature:

$$\varphi_1(\mathrm{x}) = \int_{-1}^{1} \frac{\varepsilon\, d\varepsilon}{e^{(\varepsilon/\mathrm{x})} - 1}.$$

The value of the Debye temperature θ_D can be determined by various experimental methods such as specific heat, elastic constant and diffraction measurements (Alers, 1965; Herbstein, 1961). For this study, the elastic constant method is used to calculate the Debye temperature θ_D at which nearly all modes of vibrations in a solid are excited. It is given e.g. by Alers (1965):

$$\theta_D = \frac{h}{k_B} \left(\frac{4\pi}{9}\right)^{-1/3} \rho^{\frac{1}{3}} \left(\frac{1}{v_l^3} + \frac{2}{v_t^3}\right)^{-1/3}, \qquad (3)$$

where ρ is the density of the solid and v_l and v_s the longitudinal and transverse velocities of sound waves, respectively. These velocities are given by the following relations (Ghosh & Olson, 2002; Terasaki & Yamagishi, 2011):

$$v_l = \left(B + \frac{4}{3}\,\mu\right)^{1/2} \rho^{-1/2}, \quad \text{and} \qquad (4)$$

$$v_t = \mu^{1/2} \rho^{-1/2}, \qquad (5)$$

where B is the bulk and μ the shear modulus. Eqs. (4) and (5) show the longitudinal and transverse velocities of sound waves, which are a function of the elastic properties as well as density.

For the comparisons and results presented herein, the relations of Ghosh & Olson (2002) are implemented to calculate the values of elastic properties at several temperatures. The density of the used steel at different temperatures can be calculated from the analysis given in Miettinen & Louhenkilpi (1994).

1.2. Scattering cross sections

The total microscopic neutron cross section σ_{tot} of an isotope is given by its incoherent and coherent scattering, σ_{coh}, σ_{incoh}, as well as its absorption, σ_{abs}, contributions (Vogel, 2000; Granada, 1984):

$$\sigma_{\text{tot}}(\lambda) = \sigma_{\text{coh}}(\lambda) + \sigma_{\text{incoh}}(\lambda) + \sigma_{\text{abs}}(\lambda). \quad (6)$$

The probability for a neutron to be scattered is determined by the sum of the first two cross sections. In particular, coherent (ordered) scattering appears if a regular spacing of atoms causes many scattered waves to interfere constructively. Incoherent scattering occurs due to the randomness (disorder) of the phases of the scattered waves. One distinguishes between elastic and inelastic scattering, where the latter process describes the probability for neutron interaction with the sample's crystal lattice involving the creation or destruction of phonons through loss or gain of energy by the neutron, while for elastic scattering the energy of the neutron remains constant. The total neutron attenuation coefficient considering the structure of the material can hence be written as: (Granada, 1984; Schopper *et al.*, 2000)

$$\mu_{\text{tot}}(\lambda) = \left[\bar{\sigma}_{\text{coh}} S_{\text{coh}}^{el}(\lambda) + \bar{\sigma}_{\text{incoh}} S_{\text{incoh}}^{el}(\lambda) + \sigma_{\text{total}}^{\text{inel}}(\lambda) + \sigma_{\text{abs}}(\lambda)\right]\rho_A, \quad (7)$$

where ρ_A is the atomic density and $\bar{\sigma}_{\text{coh}}$ and $\bar{\sigma}_{\text{incoh}}$ are the average cross sections calculated from the scattering length b of the nucleus: (Willis & Carlile, 2017; Dinnebier *et al.*, 2008)

$$\bar{\sigma}_{\text{coh}} = 4\pi\langle b\rangle^2, \quad (8)$$

$$\bar{\sigma}_{\text{incoh}} = 4\pi\left(\langle b^2\rangle - \langle b\rangle^2\right). \quad (9)$$

The scattering functions S describe the influence of the spatial arrangement of nuclei and their corresponding dependence on the neutron wavelength and $\sigma_{\text{total}}^{\text{inel}}(\lambda)$ the total inelastic scattering cross section:

$$\sigma_{\text{total}}^{\text{inel}}(\lambda) = \sigma_{\text{coh}}^{\text{inel}}(\lambda) + \sigma_{\text{incoh}}^{\text{inel}}(\lambda) = (\bar{\sigma}_{\text{coh}} + \bar{\sigma}_{\text{incoh}}) S_{\text{incoh}}^{\text{inel}}(\lambda). \quad (10)$$

The first component of Eq. (7) accounts for the coherent elastic component of the scattering cross section and is calculated by means of the structure factor F_{hkl} and the interplanar distance d_{hkl} for every set of *hkl* lattice planes in the crystal with $2d_{hkl} \leq \lambda$ (Granada, 1984; Binder, 1970):

$$\bar{\sigma}_{\text{coh}} S_{\text{coh}}^{el}(\lambda) = \frac{\lambda^2}{2V_0} \sum_{d_{hkl}}^{2d_{hkl}=\lambda} |F_{hkl}|^2\, d_{hkl}, \quad (11)$$

where V_0 is the unit-cell volume and F_{hkl} is the structure factor described as:

$$F_{hkl} = w_{hkl} \sum_n o_n b_n \exp\left(2\pi i\,(hx_n + ky_n + lz_n)\right) \exp\left(\frac{-B_{\text{iso},n}}{4d_{hkl}^2}\right), \quad (12)$$

where w_{hkl} is the multiplicity of a lattice plane and o_n is the site occupation factor, which relates to the atom concentration. Furthermore, the placement (x, y, z) of the nth atom is used to calculate the structure

factor with respect to a selected hkl lattice plane. In addition, the absorption cross section for cold and thermal neutrons is determined as (Windsor, 1981):

$$\sigma_{\mathrm{abs}}(\lambda) = \frac{\sigma_{\mathrm{abs}}^{2200}}{1.798\ \text{Å}}\,\lambda, \tag{13}$$

where λ is the neutron wavelength, σ_{abs}^{2200} the absorption cross section for thermal neutrons at a wavelength of $\lambda_0 = 1.798$ Å (equivalent to a neutron standard velocity $v_0 = 2200$ m·s^{-1}).
